# Proteomic Signatures of Diffuse and Intestinal Subtypes of Gastric Cancer

**DOI:** 10.3390/cancers13235930

**Published:** 2021-11-25

**Authors:** Smrita Singh, Mohd Younis Bhat, Gajanan Sathe, Champaka Gopal, Jyoti Sharma, Anil K. Madugundu, Neha S. Joshi, Akhilesh Pandey

**Affiliations:** 1 Institute of Bioinformatics, International Technology Park, Bangalore 560066, India; smrita@ibioinformatics.org (S.S.); younis@ibioinformatics.org (M.Y.B.); gajanan@ibioinformatics.org (G.S.); jyoti@ibioinformatics.org (J.S.); Madugundu.Anil@mayo.edu (A.K.M.); neha@ibioinformatics.org (N.S.J.); 2 Manipal Academy of Higher Education (MAHE), Manipal 576104, India; 3 Center for Molecular Medicine, National Institute of Mental Health and Neuro-Sciences (NIMHANS), Bangalore 560029, India; 4 Amrita School of Biotechnology, Amrita Vishwapeetham University, Kollam 690525, India; 5 Department of Pathology, Kidwai Memorial Institute of Oncology, Bangalore 560029, India; gchampaka@yahoo.co.in; 6 Department of Laboratory Medicine and Pathology, Mayo Clinic, Rochester, MN 55905, USA

**Keywords:** gastric cancer, mass spectrometry, proteomics, diffuse gastric cancer, signet ring cell carcinoma, intestinal gastric cancer

## Abstract

**Simple Summary:**

Gastric cancer comprises intestinal, diffuse and indeterminate subtypes based on histology. The intestinal and diffuse subtypes, although quite different in several respects, are still treated similarly. This study was designed to find differences at the protein level between the diffuse and intestinal subtypes using high-resolution mass spectrometry. We identified a differential proteomic signature of the two subtypes that included GREM1, BAG2, OLFM4, TRIP6 and MAGE-A9 proteins.

**Abstract:**

Gastric cancer is a leading cause of death from cancer globally. Gastric cancer is classified into intestinal, diffuse and indeterminate subtypes based on histology according to the Laurén classification. The intestinal and diffuse subtypes, although different in histology, demographics and outcomes, are still treated in the same fashion. This study was designed to discover proteomic signatures of diffuse and intestinal subtypes. Mass spectrometry-based proteomics using tandem mass tags (TMT)-based multiplexed analysis was used to identify proteins in tumor tissues from patients with diffuse or intestinal gastric cancer with adjacent normal tissue control. A total of 7448 or 4846 proteins were identified from intestinal or diffuse subtype, respectively. This quantitative mass spectrometric analysis defined a proteomic signature of differential expression across the two subtypes, which included gremlin1 (*GREM1*), bcl-2-associated athanogene 2 (*BAG2*), olfactomedin 4 (*OLFM4*), thyroid hormone receptor interacting protein 6 (*TRIP6*) and melanoma-associated antigen 9 (*MAGE-A9*) proteins. Although GREM1, BAG2, OLFM4, TRIP6 and MAGE-A9 have all been previously implicated in tumor progression and metastasis, they have not been linked to intestinal or diffuse subtypes of gastric cancer. Using immunohistochemical labelling of a tissue microarray comprising of 124 cases of gastric cancer, we validated the proteomic signature obtained by mass spectrometry in the discovery cohort. Our findings should help investigate the pathogenesis of these gastric cancer subtypes and potentially lead to strategies for early diagnosis and treatment.

## 1. Introduction

Gastric cancer is a major cause of death worldwide, particularly in Southeast Asia. Surgical resection with adjuvant chemotherapy is the preferred treatment for early gastric cancer. Recurrence occurs in up to 30–40% of patients within 5 years. Gastric cancer is a clinically heterogeneous disease with diverse histology, morphology and molecular pathogenesis. The Lauren classification and the World Health Organization (WHO) classification are the two most commonly used histologic classifications. The Lauren system classifies gastric cancer into intestinal, diffuse and indeterminate types [1], while the 2010 WHO system classifies gastric adenocarcinoma into papillary, tubular, mucinous, poorly cohesive (including signet ring cell carcinoma and other variants) and mixed adenocarcinomas [2] based on the predominant histologic pattern.

Molecular genomic studies by The Cancer Genome Atlas (TCGA) have categorized gastric cancer into four subtypes—Epstein-Barr virus positive (EBV), microsatellite instable (MSI), genomically stable (GS) and chromosomal instability (CIN) [3]. The TCGA study found an enrichment of the diffuse subtype of gastric cancer in the GS group [3]. There are several transcriptomic studies on gastric cancer, with each providing a different subtyping based on their findings. A study based on gene expression data by the Asian Cancer Research Group (ACRG) has classified gastric cancer into four subtypes—microsatellite instable (MSI), microsatellite stable and epithelial-to-mesenchymal transition (MSS/EMT), TP53 active (MSS/TP53+) and TP53 inactive (MSS/TP53-) [4]. The MSS/EMT subtype was found to have the worst prognosis and comprised mainly of diffuse gastric cancer [4]. Oh et al., performed a transcriptomic and protein analysis of gastric cancer tumor tissue using microarray and reverse phase protein arrays (RPPA), respectively [5]. They used 307 tumor tissue samples and 12 samples of surrounding non-tumor tissue for the microarray experiments and 255 samples for RPPA analysis [5]. Based on their findings, they classified gastric cancer into two molecular subtypes—mesenchymal phenotype (MP) and epithelial phenotype (EP) [5]. The MP subtype tumors were found to show a high genomic integrity and were associated with markedly poor survival and resistance to standard chemotherapy [5]. In contrast, the EP subtype tumors showed low genomic integrity and were associated with better survival rates and sensitivity to chemotherapy [5]. Lei et al., classified gastric adenocarcinoma based on gene expression data from 248 gastric tumors into three subtypes-proliferative, metabolic, and mesenchymal [6]. The proliferative subtype had high levels of genomic instability, TP53 mutations, and DNA hypomethylation, while tumors of the metabolic subtype were more sensitive to 5-fluorouracil than the other subtypes [6]. The mesenchymal subtypes showed stem cell-like features. However, they did not find strong differences in survival across the three subtypes [6].

Two recent large-scale proteomic studies focused on the diffuse subtype of gastric cancer [7,8]. Ge et al. classified the diffuse subtype of gastric cancer further into three subtypes—PX1, PX2 and PX3—based on altered proteins alone [7]. They performed proteome profiling using liquid chromatography tandem mass spectrometry (LC-MS/MS) and targeted exome sequencing of 84 paired diffuse gastric cancer samples and adjacent normal tissue [7]. They observed that PX1 and PX2 subtypes showed dysregulation of the cell cycle with PX2 featuring an additional EMT process [7]. The PX3 subtype was enriched in immune proteins, resistant to chemotherapy and exhibited the worst survival [7]. Mun et al. performed a proteogenomic study on 80 gastric cancer samples comprising of 74 diffuse, three intestinal, two mixed type, and one inflammatory myoblastic tumors [8]. They showed that early-onset gastric cancer, 92.5% of which were of the diffuse subtype in their cohort, have a mutation landscape that is different from late-onset gastric cancer [8].

Intestinal and diffuse gastric cancers show many differences in epidemiology, pathology and etiology. In the intestinal subtype, the tumor cells are arranged in tubular or glandular patterns and adhere to each other. The intestinal subtype of gastric cancer is often seen in association with intestinal metaplasia, lymphatic or vascular invasion [9,10]. This subtype of gastric cancer most commonly occurs in older men, affects the gastric antrum, shows a longer disease course and has a better prognosis [9,10]. In the diffuse subtype of gastric cancer, the tumor cells do not adhere to one another and infiltrate the stroma singly or in small clusters. On microscopy, they appear as separate, scattered tumor cells. The diffuse subtype occurs at a relatively younger age as compared to the intestinal subtype and shows a predilection for females [9]. Peritoneal metastasis is common. Precursor lesions in these cases are difficult to identify. The body of the stomach is usually affected, patients present with a shorter disease course and have a worse prognosis with the diffuse subtype [9,11]. Some studies have used the histologic classification to investigate individualized treatment in gastric cancer [12,13,14,15].

Studies published as part of the TCGA initiative and other similar large efforts have provided valuable information to help understand a wide variety of tumors. Understanding the mechanisms of carcinogenesis and its progression is necessary to improve diagnosis and prognosis. The Clinical Proteomic Tumor Analysis Consortium (CPTAC) has conducted integrated analyses, which include DNA methylation, copy number alterations (CNVs), and mRNA and protein profiling of TCGA tumor specimens from colorectal cancer, breast, and ovarian cancers [16,17,18]. These analyses provide the proteogenomic landscapes of these cancers. Mass spectrometry (MS)-based strategies to identify altered proteins in the diffuse and intestinal subtypes of gastric adenocarcinoma could help us understand its pathogenesis and progression and lead to strategies for early diagnosis and treatment of the disease.

## 2. Materials and Methods

We analyzed fresh frozen tissue samples from tumor and adjacent normal of five patients with diffuse and five patients with an intestinal subtype of gastric cancer by tandem mass spectrometry.

The study was approved by and conducted according to requirements of the Institutional Review Board and Medical Ethics Committee at Kidwai Memorial Institute of Oncology, Bangalore, India.

### 2.1. Discovery Cohort

Ten patients diagnosed with gastric cancer were included in this study for the discovery proteomics experiment; five patients had diffuse subtype of gastric cancer and five with intestinal subtype of gastric cancer. None of the patients received neoadjuvant chemotherapy. The median age of the patient cohort was 60 years (range, 48 to 66 years) with a male predominance (M:F = 4:1). Patients below 18 years of age, those treated with neoadjuvant chemotherapy and those without research consent were excluded.

### 2.2. Validation Cohort

Tissue microarrays (TMAs) were constructed using formalin-fixed paraffin embedded (FFPE) tissue blocks from 108 cases of intestinal gastric cancer and 16 cases of diffuse gastric cancer. These cases represent a larger independent set that was used for validation of candidate biomarkers and were collected from patients that were diagnosed with gastric cancer and underwent surgical resection at Kidwai Memorial Institute of Oncology, Bangalore from January, 2017 to June, 2019. None of the patients received neoadjuvant chemotherapy. The median age of the patient cohort for the intestinal subtype was 57 years (range, 27 to 82 years) and the diffuse subtype was 60 years (range, 31 to 75 years). A male predominance was seen in both the subtypes (intestinal, M:F = 2.4:1 and diffuse, M:F = 3:1).

### 2.3. Sample Collection

The tumor tissue and adjacent normal tissue were selected by a pathologist from diagnosed cases of gastric cancer. Fresh tissue samples were frozen at −80 °C until further use.

### 2.4. Protein Extraction and Normalization

Five samples of tumor tissue with their respective adjacent normal tissue (control) were taken from each of the diffuse (*n* = 10) and intestinal (*n* = 10) subtypes. Each experiment was carried out separately for the intestinal and diffuse subtypes. Thus, five pairs of tumor and adjacent normal tissue (control) from the diffuse subtype was used for one experiment and five pairs of tumor and adjacent normal tissue (control) from the intestinal subtype was used in the subsequent experiment.

The fresh frozen tissue samples were homogenized individually in liquid nitrogen using a mortar and pestle. Proteins from these tissues were extracted in urea lysis buffer (9 M urea, 20 mM HEPES, 1 mM sodium orthovanadate, 1 mM β-glycerophosphate, and 2.5 mM sodium pyrophosphate) heated at 95 °C for 5 min. This was followed by probe sonication using a Sonifer cell disruptor (Branson 150, Emerson Electric Co., St. Louis, MO, USA). The lysates were subjected to centrifugation at 10,000× *g* for 15 min at 4 °C to remove cell debris. The supernatant from each tissue lysate was collected and protein estimation was carried out by bicinchoninic acid (BCA) assay (Pierce; Waltham, MA, USA) in accordance with the manufacturer’s protocol. Protein samples were normalized based on protein amounts, as verified on 10% sodium dodecyl sulfate-polyacrylamide gel electrophoresis (SDS-PAGE).

Lysates from each sample equivalent to 500 µg of protein were taken for reduction and alkylation of cysteine residues. Reduction and alkylation of cysteine residues were carried out using a final concentration of 10 mM dithiothreitol (DTT) at 60 °C for 20 min; and 20 mM iodoacetamide (IAA) at room temperature for 30 min in the dark, respectively. The proteins were precipitated using ice cold acetone. The precipitated proteins were dissolved in a solution of 6 M urea in 50 mM Triethyl Ammonium Bicarbonate (TEAB) and was used for in-solution digestion.

### 2.5. In-Solution Digestion

In-solution digestion was carried out with 500 µg of protein from each sample. Digestion of the samples was carried out using Promega Lys-C (sequencing grade) in the ratio of 1:100 (enzyme:protein) and samples were incubated at room temperature for 4 h followed by digestion with Promega Sequencing Grade Modified Trypsin in the ratio of 1:20 (enzyme: protein) at 37 °C overnight at 1000 rpm in a thermomixer. Samples were then acidified with an aqueous solution of 1% formic acid to stop the reaction and were desalted using Sep-Pak C_18_ cartridges (Waters, Milford, MA, USA). Eluted samples were dried in a speed vacuum at 35 °C. The dried samples were reconstituted in 110 µL of 100 mM TEAB.

### 2.6. TMT-Labeling: Diffuse and Intestinal Subtypes

Resulting peptides from the diffuse and intestinal subtypes and their adjacent normal tissue were labeled using 10-plex Tandem Mass Tag (TMT) labels as per the manufacturer’s instructions (Catalog # 90110, Thermo Fisher Scientific, Waltham, MA, USA) for each subtype. The TMT-labeling reaction was quenched using 5% hydroxylamine prior to pooling the samples. The pooled samples were dried in a speed vacuum at 35 °C.

### 2.7. Basic pH Reversed-Phase Liquid Chromatography (bRPLC)

Pooled TMT-labeled samples were fractionated by basic pH reversed-phase chromatography (bRPLC) into 96 fractions. The TMT-labeled peptide mixture was resuspended in 110 µL of bRPLC solvent A (5 mM ammonium formate in water, pH 8.5) and fractionated by bRPLC chromatography on a C_18_, 250 mm × 4.6 mm column, 5 µm, XBridge (Waters, Milford, MA, USA) by employing an increasing gradient of bRPLC solvent B (5 mM ammonium formate in 90% acetonitrile, pH 8.5) on a Vanquish UHPLC (Thermo Fisher Scientific, MA, USA). A total of 96 fractions were collected, which were then concatenated into 12 fractions and dried in a speed vacuum. These 12 fractions were desalted using C_18_ stage tips (3M Empore C_18_ extraction disks, Fisher Scientific, Waltham, MA, USA) prior to analysis on the mass spectrometer.

### 2.8. TMT-Labeled Quantitative Proteomics for Global Profiling of Protein Expression Levels

**Figure 1:**
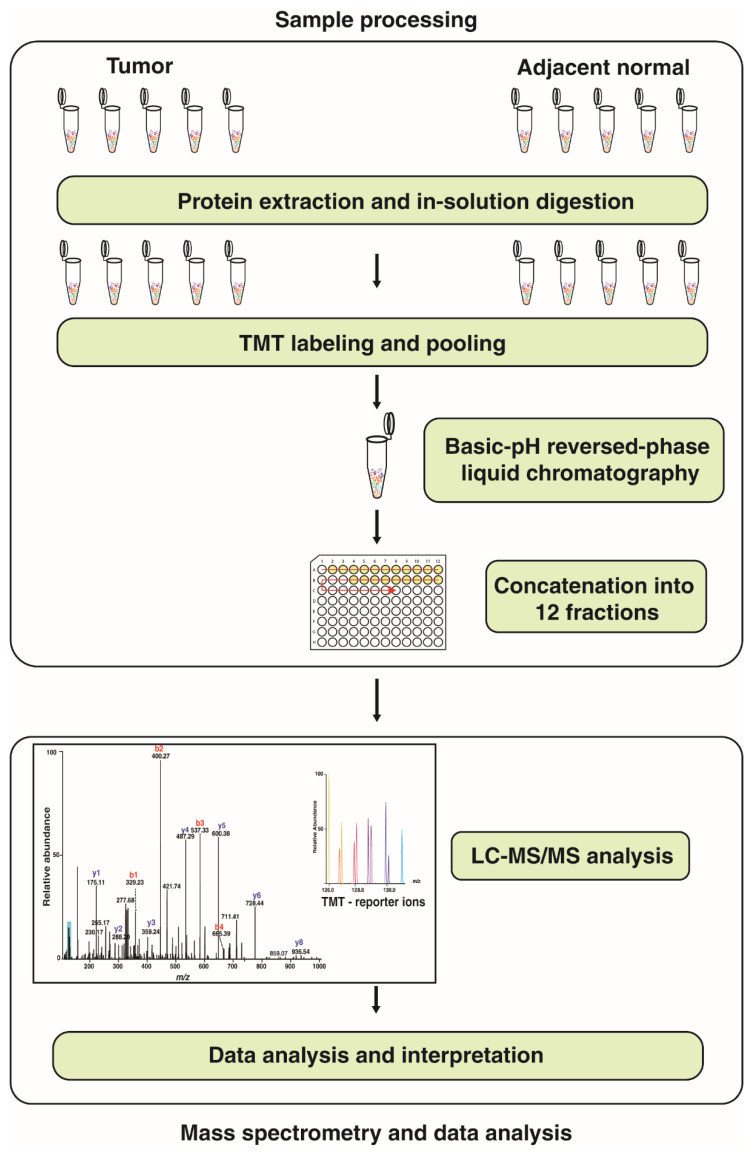
A schematic of the workflow used to study the proteome in the diffuse and intestinal subtypes of gastric cancer. Paired tumor and adjacent normal tissue from each subtype were analyzed using TMT-labeling to quantify proteins using LC-MS/MS.

Quantitative proteomic analysis was carried out using five paired samples of tumor tissue and adjacent normal tissue, respectively, for each gastric cancer subtype. TMT-labeled samples were analyzed on a Q Exactive HF-X hybrid quadrupole-Orbitrap mass spectrometer (Thermo Scientific, San Jose, CA, USA) coupled to a UHPLC (UltiMate 3000, Thermo Fisher Scientific, Waltham, MA, USA). Peptides were reconstituted in 0.1% formic acid and loaded on a trap column (Thermo Scientific Acclaim PepMap 100 C_18_ LC column, 75 µm × 2 cm) for resolution. The nano column was eluted with a multi-step gradient of 4–90% solvent B (Solvent A: 0.1% formic acid in water; Solvent B: 95% acetonitrile and 0.1% formic acid in water) over 70 min with a flow rate of 300 nL/min with a total run time of 120 min. The MS data acquisition was carried out from 400–1600 m/z range using an Orbitrap mass analyzer. The automatic gain control (AGC) target was set to 500,000 with an ion injection time of 50 ms and a dynamic exclusion of 30 s. The Q Exactive HF-X mass spectrometer was operated in the data-dependent acquisition (DDA) mode selecting the top 20 most intense precursors from each scan for fragmentation. Precursor ions were fragmented using high collision dissociation (HCD) and were analyzed using an Orbitrap mass analyzer. For precursor ions, the AGC target was set to 100,000 with an ion injection time of 100 ms. The workflow is summarized in Figure 1.

### 2.9. Data Analysis

Proteome Discoverer 2.3 software (Thermo Fisher Scientific, Bremen, Germany) was used to perform database searches against the Human RefSeq protein database (Version 92) using a SEQUEST-HT search algorithm. The workflow that was used for the searches included spectrum selector, SEQUEST search nodes and a peptide validator. The search parameters involved were carbamidomethylation at cysteine residues (+57.021 Da), TMT 10-plex (+229.163 Da) modification at N-terminus of peptide and C-terminus of lysine were set as fixed modifications and oxidation of methionine (+15.995 Da) was set as a dynamic modification. MS and MS/MS mass tolerances were set to 10 ppm and 0.05 Da, respectively. Trypsin was specified as protease and a maximum of two missed cleavages were allowed. An FDR of 1% was applied at protein and peptide levels as a cut-off value for reporting identified peptides.

### 2.10. Statistical Analysis

Data generated from both experiments was analyzed using the Perseus software package (Version 1.6.2.2, Max Planck Institute of Biochemistry, Martinsried, Germany). The number of significantly dysregulated proteins was estimated by applying the paired two-tailed t-test with a *p*-value less than 0.05 and a greater than 2-fold-change in expression between groups (tumor vs. adjacent normal).

### 2.11. Bioinformatics Analysis

The list of gene symbols of differentially expressed proteins along with their fold-change values in the diffuse and intestinal subtypes were uploaded to the Ingenuity Pathway Analysis (IPA) for core analysis (QIAGEN Inc.). IPA-generated protein interactions, pathways, functional and upstream regulatory networks. The following parameters were used to perform the core analysis: the expression fold-change values were set as the type of core analysis; to generate networks, direct and indirect relationships were considered and endogenous chemicals with 35 molecules per network were included for network prediction. A total of 25 networks were enabled per analysis. Other parameters set in this analysis were Homo sapiens as species and all human tissues and primary cells.

Log_2_ fold-change ≥1 for upregulated and ≤−1 for downregulated proteins were the cutoff values applied to all datasets included. *p*-value < 0.05 was considered significant. The Z-score was used to assign the predicted possible upstream regulators of the differentially expressed proteins as either inhibited or activated. IPA identified the top canonical pathways and molecular networks, which were ranked based on their significance (*p*-values calculated using the right tailed Fisher’s exact test) from our input dataset.

### 2.12. Immunohistochemistry

Formalin-fixed paraffin-embedded (FFPE) blocks of intestinal and diffuse gastric cancer were obtained from the Department of Pathology, Kidwai Memorial Institute of Oncology, Bangalore, India. Tissue microarrays (TMA) were constructed using FFPE blocks from 108 cases of intestinal gastric cancer and 16 cases of diffuse gastric cancer. These cases represent a larger independent set that was used for validation of candidate biomarkers. The samples were collected from patients that were diagnosed with gastric cancer and underwent surgical resection at Kidwai Memorial Institute of Oncology, Bangalore from January, 2017 to June, 2019. None of the patients received neoadjuvant chemotherapy. 2 mm cores were taken in duplicate from the areas representative of the tumor determined from hematoxylin and eosin (H&E) stained sections of the corresponding blocks and embedded into Quick-Ray TMA recipient blocks.

3 µm sections were cut from the tissue microarray blocks on to TOMO, IHC Adhesive Hydrophilic Slides (Matsunami, Japan) and labeled with the block ID. These slides were baked overnight at 65 °C before deparaffinization. Deparaffinization of the tissue sections was done in xylene (2 × 10 min) followed by absolute alcohol (5 min) and 95% alcohol (5 min). Blocking of endogenous peroxidases was done by treating the sections with a 3% *v*/*v* solution of hydrogen peroxide in methanol for 20 min. The sections were then washed in 70% alcohol (2 min) followed by 0.05M tris-buffered saline (TBS), pH 7.6.

Tris-ethylenediaminetetraacetic acid (Tris-EDTA) buffer, pH 9.0, was used for antigen retrieval in a pressure cooker for 20 min. The slides were allowed to cool down to room temperature before transferring them to TBS. A solution of 0.25% casein in phosphate buffered saline (PBS) (Dako North America Inc., Carpinteria, CA, USA) was used to block endogenous biotin, which was applied to the tissue sections for 30 min. The primary antibody was applied to the TMA sections. The primary antibodies used were anti-NOSTRIN (1:50, Santa Cruz Biotechnology Inc., Dallas, TX, USA), anti-LIPF (1:250, AbCam, Waltham, MA, USA), anti-GREM1 (1:100, AbCam, Waltham, MA, USA), anti-BAG2 (1:100, AbCam, Waltham, MA, USA), anti-OLFM4 (1:50, AbCam, Waltham, MA, USA), anti-TRIP6 (1:100, Santa Cruz Biotechnology Inc., Dallas, TX, USA), anti-P3H3/LEPREL2 (1:100, LSBio, Seattle, WA, USA), anti-IGFBP7 (3 µg/mL, LSBio, Seattle, WA, USA), and anti-MAGE-A9 (1:100, LSBio, Seattle, WA, USA). The sections were then incubated overnight at 4°C with the primary antibodies. Following incubation, the slides were washed in TBS (2 changes, 5 min each). The secondary antibody was horseradish peroxidase (HRP) conjugated anti-mouse/anti-rabbit polyclonal IgG antibody (Dako North America Inc., Carpinteria, CA, USA). The slides were treated with the secondary antibody for 30 min and then washed in TBS (2 washes, 5 min each). A 1% solution of 3,3′-diaminobenzidine (DAB) peroxidase substrate was applied to the sections for 5 min. The slides were washed in distilled water (two washes, 2 min each). They were then counterstained with Harris’ hematoxylin for 30 s and washed in running tap water for 2 min. Each staining run included positive and negative external controls.

For dehydration and clearing, the slides were washed in two jars placed in sequential order containing 95% alcohol for 2 min and absolute alcohol (2 changes for 3 min each). Xylene was used for clearing (2 changes, 5 min each). DPX and appropriate cover slips were used for mounting the sections. These mounted sections were incubated at 50 °C for 30 min for drying. A pathologist examined the slides for the intensity and distribution of staining in both diffuse and intestinal subtypes and scored them using the H-score.

### 2.13. Correlation of Transcriptomic and Proteomic Data

We wanted to look at the correlation between the mRNA expression in the TCGA gastric cancer data [3] and the proteins reported in this study. RNA-Seq data were extracted from the Xena portal for the TCGA TARGET GTEx project for diffuse and intestinal subtypes of gastric adenocarcinoma (Table 1).

RNA-Seq expression data of the genes corresponding to the differentially expressed genes in protein data (Table 2) were set apart and used for correlation analysis. The expression data were in the form of log_2_ (norm_count+1), which was converted to read counts for input in DESeq2. The log_2_ fold-change values for protein data was taken for each subtype of gastric cancer from our findings. Median value was calculated for genes, which had multiple fold-change values. The log_2_ fold-change for RNASeq expression data was calculated using DESeq2. The fold-change values are provided in Supplementary Table S1.

Fold-change values for genes overlapping in both the datasets were considered for correlation analysis. Pearson correlation (r) was calculated using Cor function in R.

### 2.14. Survival Analysis for GREM1, BAG2, TRIP6, OLFM4 and MAGE-A9

We wished to study the impact of the expression of *GREM1, BAG2, TRIP6, OLFM4* and *MAGE-A9* on the survival of gastric cancer patients with either diffuse or intestinal subtype of gastric cancer. We used the mRNA expression data and the duration of survival for each of these proteins from the TCGA data to generate Kaplan–Meier plots. The Kaplan–Meier plots were generated using SPSS software (Version 28.0.0.0 (190), IBM, Armonk, NY, USA) for *GREM1, BAG2, TRIP6, OLFM4* and *MAGE-A9*. The log-rank test was used to calculate the *p*-value. The parameters used were: (1) Time: Days until death (extracted from clinical data provided in TCGA GDC portal), (2) Status: Gene expressions, and (3) Factor: Type of Gastric cancer—Diffuse or Intestinal. The data used for plotting the Kaplan–Meier plots is provided in Supplementary Table S2.

## 3. Results

### 3.1. Proteomic Analysis of Diffuse and Intestinal Subtypes of Gastric Cancer

Five paired samples of gastric tumor and adjacent normal tissues were taken for each of the diffuse and intestinal subtypes. The baseline demographic and disease characteristics of these patients are given in Table 3. Protein was extracted from the tumor and adjacent normal tissues and subjected to in-solution digestion using trypsin. The peptides from each sample were labeled with TMT 10-plex labels and pooled. The pooled samples were then analyzed by LC-MS/MS on a Q Exactive HF-X hybrid quadrupole-Orbitrap mass spectrometer.

**Figure 2:**
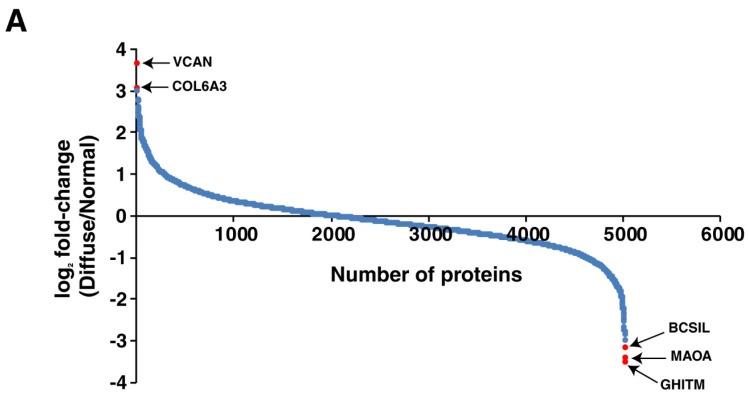

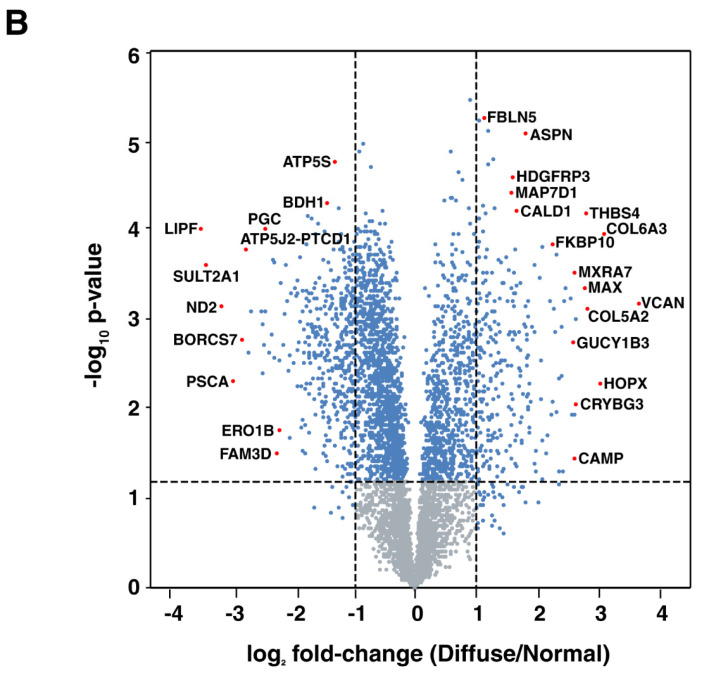
Waterfall plot showing the distribution of the significantly upregulated and downregulated proteins (*p*-value < 0.05) in (**A**) and a volcano plot showing the distribution of proteins identified in the diffuse subtype of gastric cancer in (**B**).

**Figure 3:**
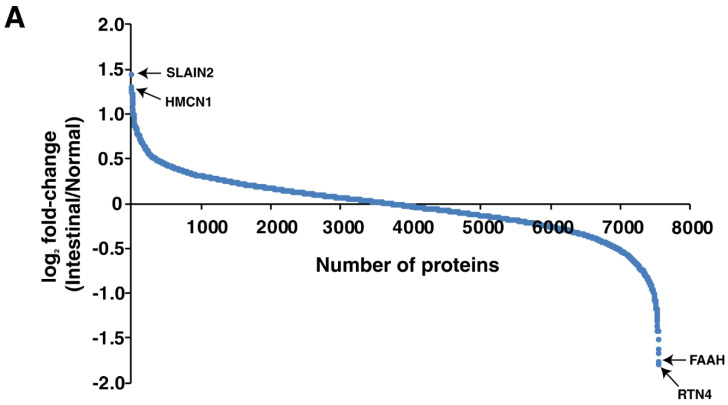

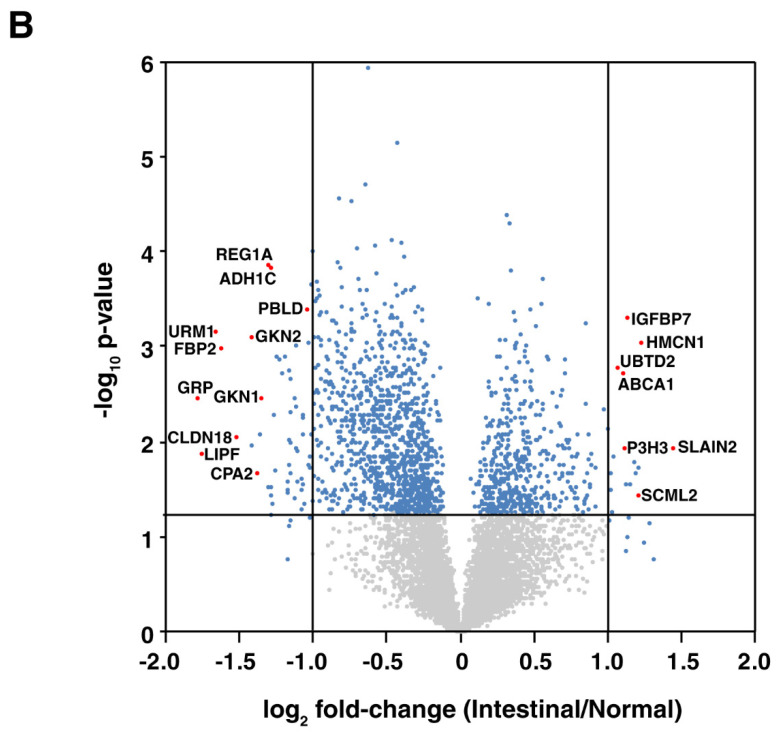
Waterfall plot showing the distribution of the significantly upregulated and downregulated proteins (*p*-value < 0.05) in (**A**) and a volcano plot showing the distribution of proteins identified in the intestinal subtype of gastric cancer in (**B**).

TMT-based quantitative proteomic analysis of the tumor and adjacent normal resulted in the identification of 4846 proteins in the diffuse subtype, of which 255 proteins were upregulated and 372 were downregulated by ≥2 fold in the tumor. In the intestinal subtype, TMT-based quantitative proteomic analysis of the tumor and adjacent normal resulted in the identification of 7448 proteins. Of these 7448 proteins, 15 were upregulated and 56 were downregulated by ≥2 fold in the tumor. The distribution of the differentially expressed proteins in the diffuse subtype is shown in Figure 2A as a waterfall plot and Figure 2B as a volcano plot. Similarly, the distribution of the differentially expressed proteins in the intestinal subtype is shown as a waterfall plot and a volcano plot in Figure 3A,B, respectively.

**Figure 4:**
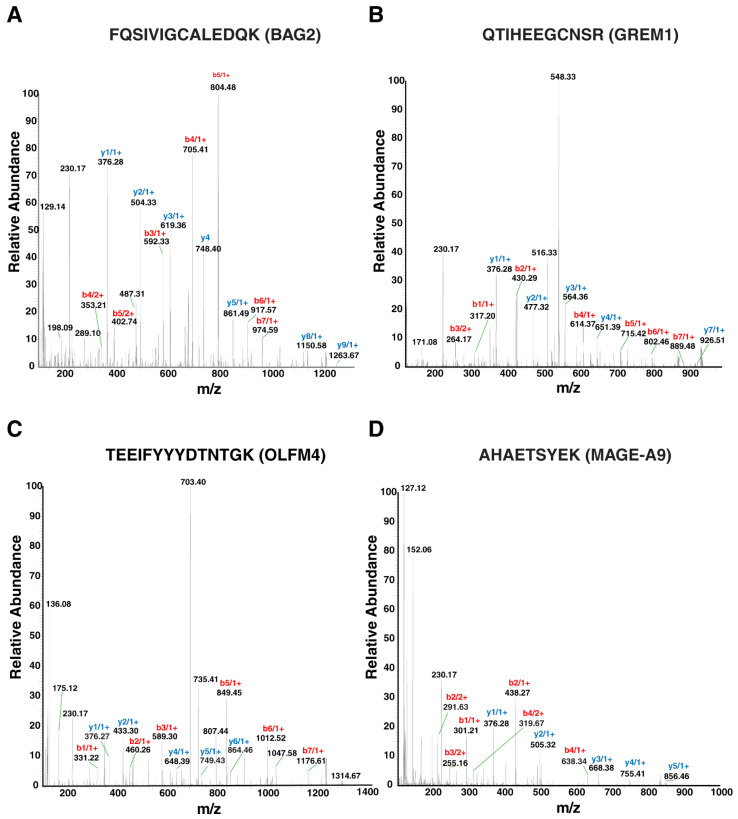
Peptides identified by LC-MS/MS analysis of candidate proteins—BAG2, GREM1, OLFM4 in diffuse, and MAGE-A9 in intestinal subtypes of gastric cancer. Representative MS/MS spectra of the peptides—(**A**) FQSIVIGCALEDQK identified in BAG2, (**B**) QTIHEEGCNSR identified in GREM1, (**C**) TEEIFYYYDTNTGK identified in OLFM4 in diffuse subtype of gastric cancer and (**D**) AHAETSYEK identified in MAGE-A9 in intestinal subtype of gastric cancer.

A partial list of the differentially expressed proteins in diffuse and intestinal subtypes of gastric cancer are given in Table 4 and Table 5 respectively. Representative MS/MS spectra of a few of the peptides identified by LC-MS/MS analysis—BAG2, GREM1, OLFM4 and MAGE-A9 are shown in Figure 4. A complete list of the proteins identified in both the intestinal and diffuse subtypes of gastric cancer along with their fold-change values are given in Supplementary Table S3.

### 3.2. Functional Enrichment Analysis

Protein–protein interactions mediated by signaling networks are thought to play a role in communicating changes in protein expression to changes in biological function. Ingenuity Pathway analysis (IPA) (Version 52912811) of the differentially expressed proteins in the diffuse subtype revealed alterations in mitochondrial function, oxidative phosphorylation, sirtuin signaling pathway, tricarboxylic acid (TCA) cycle and GP6 signaling pathway, which were the top five canonical pathways identified (Supplementary Table S5). Oxidative phosphorylation is commonly involved in cancer and has been implicated in gastric cancer [19,20]. Decreased oxidative phosphorylation activity has been proposed to be related to reduced mitochondrial DNA content or increased mutations in mitochondrial DNA [21]. This is supported by altered mitochondrial function in a diffuse subtype. The TCA cycle is necessary for the generation of ATP and other molecules involved in lipid and DNA synthesis. Decreased activity of key TCA cycle enzymes like isocitrate dehydrogenase (IDH), succinate dehydrogenase (SDH), malate dehydrogenase (MDH) and α-ketoglutarate dehydrogenase (α-KGDH) have been described in various cancers including gastric cancer [22,23], which is consistent with our findings. Upregulation of the sirtuin signaling pathway was seen in the diffuse subtype of gastric cancer. Several studies have shown SIRT1 to be upregulated and to inhibit tumor growth and metastasis in gastric cancer, suggesting that SIRT1 may act as a tumor suppressor [24,25,26]. The upregulation of SIRT1 in gastric cancer could be the result of a feedback mechanism that reduces the damaging effects of STAT3 signaling.

In the intestinal subtype, the top five canonical pathways included ethanol degradation II, serotonin degradation, noradrenaline and adrenaline degradation, retinoate biosynthesis and tryptophan degradation (Supplementary Table S5). In the ethanol degradation II pathway, ethanol enters the endoplasmic reticulum, where cytochrome P450 2E1 (CYP2E1) oxidizes and returns the acetaldehyde to the cytoplasm, which enters the mitochondrial compartment where it is converted to acetate by mitochondrial aldehyde dehydrogenase [27]. Decreased activity of the ethanol degradation II pathway causes accumulation of acetaldehyde, which has been shown to cause gastric cancer due to its DNA damaging effects [28,29]. *H. pylori* infection, which is associated with the intestinal subtype of gastric cancer, causes achlorhydric atrophic gastritis. High gastric acetaldehyde production has been reported in patients with achlorhydric atrophic gastritis [30]. Serotonin has been shown to stimulate growth of tumor cells in several cancers, including prostate carcinoma, bladder carcinoma, small-cell lung carcinoma, colorectal carcinoma, hepatocellular carcinoma and cholangiocarcinoma [31]. Fluoxetine, a selective serotonin reuptake inhibitor, was shown to inhibit the growth of cancer cells by inducing apoptosis in gastric cancer cells through various signaling pathways [32]. Downregulation of the serotonin degradation pathway might lead to accumulation of serotonin, thereby promoting growth of tumor cells. Adrenaline and noradrenaline are catecholamines. Catecholamine activity has been shown to upregulate MMP-7 levels through the β2-receptor adrenergic signaling pathway in gastric cancer cells [33]. Overexpression of MMP-7 is frequently seen in premalignant and malignant gastric lesions [34,35]. It has also been associated with invasion, lymph node metastasis, peritoneal dissemination and survival of gastric cancer patients [36]. Downregulation of the adrenaline and noradrenaline degradation pathway could enhance the activity of these catecholamines and their oncogenic role.

### 3.3. IPA Network Analysis in Gastric Cancer

We performed network analysis using IPA on the list of differentially expressed proteins in both the diffuse and intestinal subtypes of gastric cancer. IPA network analysis of the diffuse subtype showed upregulation of proteins like HSPB1 [37,38], KCTD [39,40,41,42], TPM3 [43,44,45,46], PDLIM7 [47,48,49], LUZP1 [50], ACTN1 [51,52], and CFL2 [53], which have oncogenic properties in the topmost network (Supplementary Figure S1). These proteins are involved in promoting tumor growth, invasion and resistance to anti-tumor therapy in various cancers including gastric cancer. Downregulation of proteins involved in the mitochondrial respiratory chain like NFU1 [54,55], ACO2 [56], PDHA1 [57], and proteins like DECR1 [58], CNDP2 [59], and SPINT1 [60,61], which have a tumor suppressive role in different cancers were noted.

The topmost molecular network in the intestinal subtype on IPA network analysis showed increased expression of SULF1 [62], HGF [63,64], SPARC [65,66,67], SERPINH1 [68], and IGFBP7 [69,70], which have been shown to contribute to the growth and survival of tumor cells, tumor invasion, metastasis, and poor survival in gastric cancer (Supplementary Figure S2). AKR1B10 [71], GKN1 [72,73], TFF2 [74,75], SULT1E1, ADH1C, ADHFE1, ADH7, REG3A, LIPF, ATP4A, ATP4B, and PGC were found to show decreased expression. These proteins have been shown to play a role in tumor suppression and inhibit gastric carcinogenesis and metastasis [76,77,78,79,80,81,82,83].

### 3.4. Increased Expression of GREM1, BAG2, OLFM4, TRIP6 in the Diffuse Subtype and MAGE-A9 in the Intestinal Subtype

**Figure 5:**
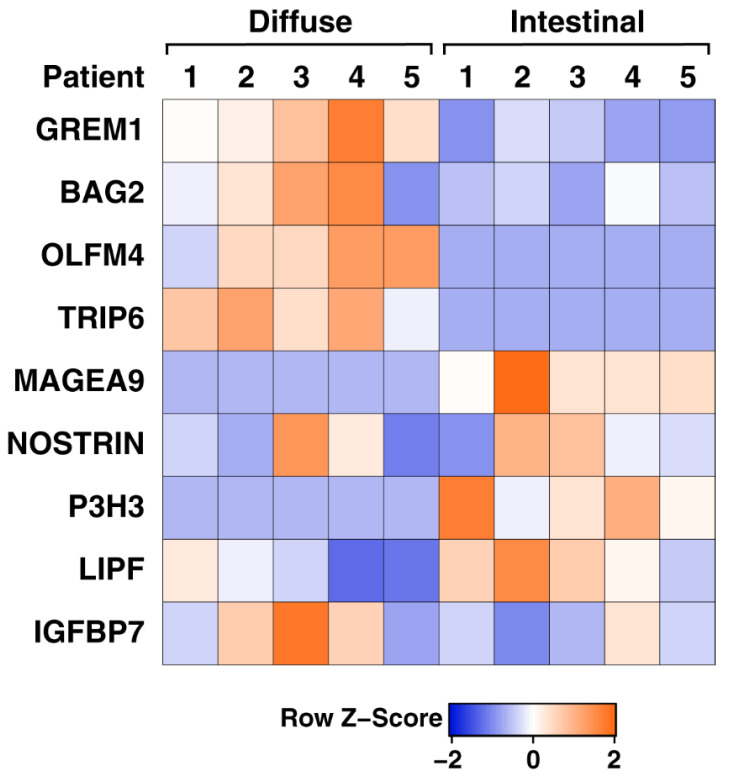
Heatmap of the candidate proteins shortlisted for validation in the diffuse and intestinal subtypes of gastric cancer. The heatmap is color-coded by correlation according to the color legend. Intensity and directions of correlations are indicated at the bottom of the heat map (red, positively correlated, blue, negatively correlated).

We selected nine proteins—NOSTRIN, GREM1, BAG2, OLFM4, TRIP6, IGFBP7, P3H3, LIPF, and MAGE-A9 based on their differential expression in the diffuse and intestinal subtypes and because they had not been studied in the diffuse and intestinal subtypes of gastric cancer. We compared the fold-change values for these nine proteins in both the subtypes and found a change in opposing directions as illustrated in the heatmap (Figure 5). In order to validate our mass spectrometric findings, we performed IHC using antibodies directed against these nine proteins—NOSTRIN, GREM1, BAG2, OLFM4, TRIP6, IGFBP7, P3H3, LIPF, and MAGE-A9 in both the subtypes on TMAs containing a total of 108 cases of intestinal subtype of gastric cancer and 16 cases of the diffuse subtype of gastric cancer.

**Figure 6:**
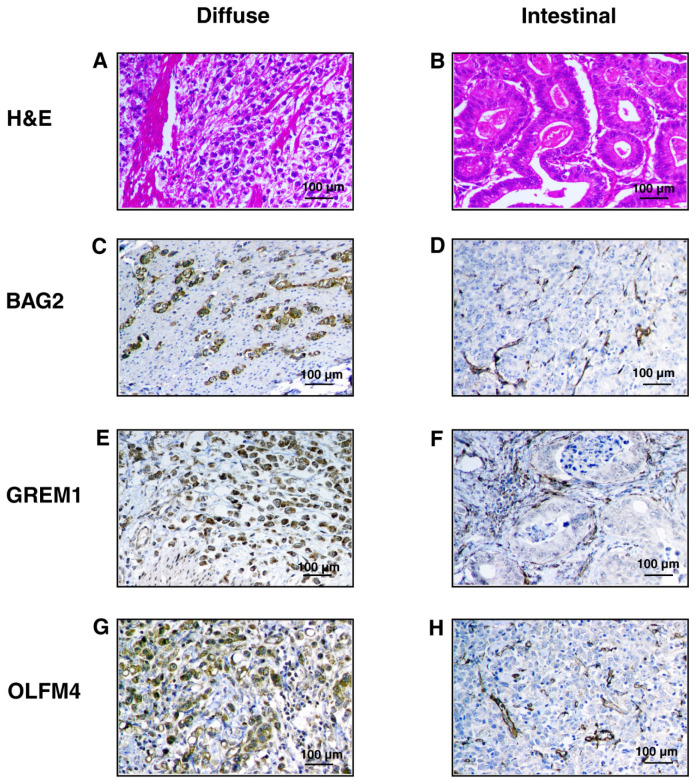

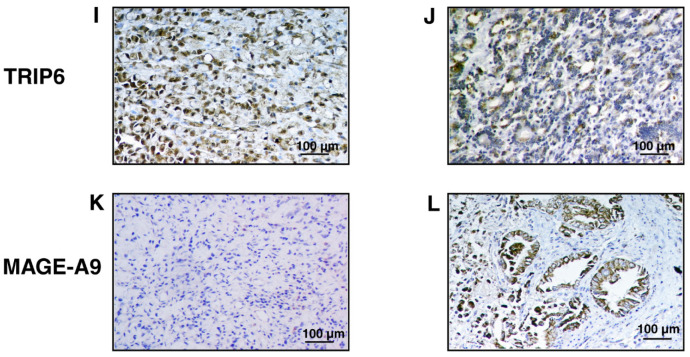
Representative photomicrographs of hematoxylin and eosin (H&E) stained sections show poorly cohesive tumor cells in a fibrous stroma in the diffuse subtype of gastric cancer (**A**) and tumor cells arranged in tubules in the intestinal subtype (**B**) IHC staining with anti-BAG2, anti-GREM1, anti-OLFM4, and anti-TRIP6 antibodies showed moderate to strong cytoplasmic staining in the tumor cells in the diffuse subtype of gastric cancer in (**C**,**E**,**G**,**I**) represented by brown color and weak to no staining in the tumor cells in the intestinal subtype in (**D**,**F**,**H**,**J**). IHC staining with anti-MAGEA9 antibodies showed moderate, granular cytoplasmic staining in the tumor cells in the intestinal subtype in (**L**) but no staining was observed in the tumor cells in the diffuse subtype in (**K**).

The immunostaining was evaluated by a pathologist and scored using the semi-quantitative H-score to calculate the sum of the percentage and intensity of positively stained tumor cells in the tumor component [84]. Each tumor specimen was scored once, with an average of two FFPE cores representing the same tumor from the same patient. A tumor sample with an H score ≥ 50 was considered positive. The final H-score obtained for a marker in each subtype was the average of all the scores for all the samples (Supplementary Table S4). IHC showed strong cytoplasmic staining in diffuse gastric cancer cells for GREM1 (H-score = 201.6), BAG2 (H-score = 169.4), TRIP6 (H-score = 175.2), and OLFM4 (H-score = 187.4) but staining was negative to weak in the intestinal subtype as seen by an H-score of <50 for all the four proteins. Moderate, granular cytoplasmic staining was seen in intestinal gastric cancer cells for MAGE-A9 (H-score = 144.8), but staining was negative in the diffuse subtype for MAGE-A9 (H-score < 50). Representative H&E stained and IHC photomicrographs of GREM1, BAG2, TRIP6, OLFM4, and MAGE-A9 are shown in Figure 6.

These findings are concordant with our findings on proteomic analysis of both the diffuse and intestinal subtypes of gastric cancer. We did not find any difference in the staining distribution and intensity between the diffuse and intestinal subtypes on IHC for NOSTRIN, IGFBP7, P3H3 and LIPF.

### 3.5. Correlation of Tumor Proteomes with mRNA Expression in Gastric Cancer

**Figure 7:**
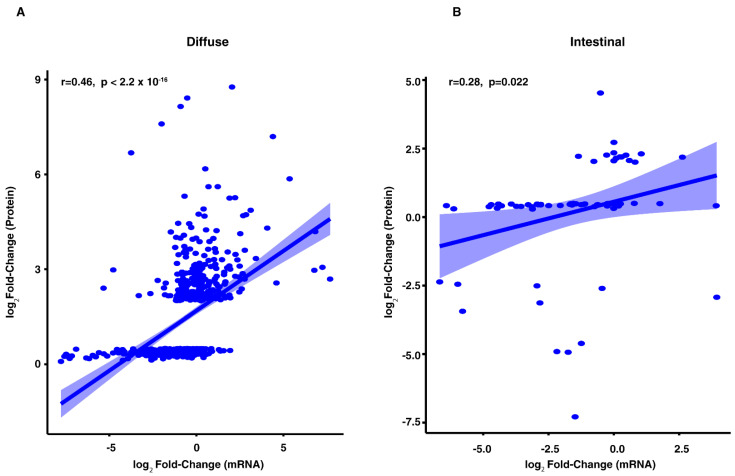
Comparison of proteomic and transcriptomic data. The log_2_ (fold-change) in mRNA gene expression is plotted against the log_2_ (fold-change) in protein abundance in diffuse (**A**) or intestinal (**B**) subtypes of gastric cancer as shown.

To investigate the correlation between the proteome and the transcriptome in the diffuse and intestinal subtypes of gastric cancer, we used RNA-Seq data from the TCGA cohort [3] corresponding to the proteins identified in this study. We found a positive correlation in both the subtype, which was modest for the diffuse subtype (r = 0.46, *p*-value < 2.2 × 10^−16^) (Figure 7A), and weak for the intestinal subtype (*r* = 0.28, *p*-value = 0.02) (Figure 7B). Our findings are similar to previous studies conducted on mRNA and protein correlation where no strong positive correlation was observed [85,86].

### 3.6. GREM1, BAG2, TRIP6, OLFM4 and MAGE-A9 Expression Do Not Show Association with Survival in Gastric Cancer

Survival analysis for *GREM1, BAG2, TRIP6, OLFM4* and *MAGE-A9* mRNA expression from the TCGA dataset from patients with the diffuse or intestinal subtypes of gastric cancer did not show any statistically significant difference. This was shown by the log-rank test for *GREM1* (*p*-value = 0.27), *BAG2* (*p*-value = 0.37), *TRIP6* (*p*-value = 0.25), *OLFM4* (*p*-value = 0.46) and *MAGE-A9* (*p*-value = 0.74). The Kaplan–Meier survival curves for each of these five genes are depicted in Supplementary Figure S3.

## 4. Discussion

Gastric cancer is one of the most common malignancies globally. Patients with gastric cancer have a high incidence of metastasis and a high mortality rate [87]. They are often diagnosed late, undergo radical gastric resection and have a poor 5-year survival rate [87]. Although the exact cause of gastric cancer is unclear, it is a multi-step, multi-factorial disease. The mechanism of gastric carcinogenesis is poorly understood and has not previously, to our knowledge, been explored between the diffuse and intestinal subtype using a proteomic approach. Mass spectrometry-based global proteomic approaches allow cataloguing of proteins and predictive modeling of associated networks in biological samples. Thus, we conducted a proteomic analysis of the diffuse and intestinal subtypes of gastric cancer to discover proteins that could provide novel mechanistic insights into carcinogenesis of each of these two subtypes.

Both the large-scale proteomic studies on gastric cancer conducted by Ge et al. and Mun et al., focused mainly on the diffuse subtype of gastric cancer [7,8]. Both were proteogenomic studies and with a fairly large number of samples (~80 paired tumor and adjacent normal tissue samples). This is conceptually similar to our study where we also used paired tumor and adjacent normal tissue. However, these previous studies did not include a set of samples for validation as in our study. Mun et al. looked at the exome, transcriptome, proteome and phosphoproteome of gastric cancer samples [8], their major aim being to study the molecular changes in early-onset gastric cancer. Thus, their samples consisted of three intestinal, two mixed type, and one inflammatory myoblastic tumors in addition to 74 diffuse gastric cancer samples. They were able to classify early-onset gastric cancer into four subtypes based on mRNA, phosphoproteome and N-glycoproteome data [8]. Ge et al. aimed to classify diffuse gastric cancer into molecular subtypes based on patient outcomes and treatment by employing targeted exome sequencing and protein analysis [7]. These studies differ from our study in that we aimed to look at molecular differences at the protein level between intestinal and diffuse subtypes of gastric cancer and not just in the diffuse subtype as was the case with these two studies.

IPA showed alterations in mitochondrial function, oxidative phosphorylation, sirtuin signaling pathway, tricarboxylic acid (TCA) cycle and GP6 signaling pathway as the top five canonical pathways identified in the diffuse subtype. In the intestinal subtype, the top five canonical pathways included ethanol degradation II, serotonin degradation, noradrenaline and adrenaline degradation, retinoate biosynthesis and tryptophan degradation. Alterations in these pathways have been shown to promote tumorigenesis, tumor growth, invasion and metastasis in different cancers including gastric cancer. Dysfunction in oxidative phosphorylation, accumulation of toxic metabolites, and generation of free radicals are known to promote carcinogenesis through DNA damage.

Network analysis using IPA of the differentially expressed proteins in the diffuse subtype showed increased expression of proteins like HSPB1, KCTD, TPM3, PDLIM7, LUZP1, ACTN1, and CFL2; and decreased expression of proteins involved in the mitochondrial respiratory chain like NFU1, ACO2, PDHA1, and tumor suppressors like DECR1, CNDP2, and SPINT1 in the topmost network. Mitochondrial dysfunction causes disturbances in cellular energy production, which supports the metabolic reprogramming of cancer cells. It also triggers carcinogenic changes mediated by reactive oxygen species (ROS), Ca^2+^ ions, or other metabolites released by mitochondria.

DNA mutations or enzyme defects in mitochondria have been shown to increase production of mitochondrial ROS [88]. High ROS levels promote cancer progression by activating signaling pathways that regulate antioxidant systems and metabolic adaptation. They also play a role in cellular proliferation, apoptosis, resistance to treatment, and tumor invasion [88]. Increased expression of tumor suppressors like EPB41L3 [89,90], and downregulation of oncogenic proteins like FGD6 [91] and CSRP1 [92] could be the result of compensatory mechanisms.

Network analysis of the differentially expressed proteins in the intestinal subtype showed increased expression of proteins with an oncogenic role (SULF1, HGF, SPARC, SERPINH1, IGFBP7) and decreased expression of proteins with a tumor suppressive function (AKR1B10, GKN1, TFF2, ADH1C, ADHFE1, ADH7, REG3A, LIPF, ATP4A, ATP4B, PGC) in gastric cancer. Both of these are favourable for tumor progression and metastasis. HGF-c-Met pathway plays a pivotal role on the growth, survival and invasiveness of GC [63,64]. High expression of SPARC is associated with disease progression and poor survival in gastric cancer [65,66,67]. SERPINH1 has been shown to play a role in epithelial-mesenchymal transition (EMT) and metastasis in gastric cancer [68]. GKN1 is a protein expressed by the mucosal cells of the antrum and fundus of the stomach. It maintains gastric homeostasis, inhibits inflammation and is a tumor suppressor. *H. pylori*-infected gastric mucosa or inflammation in the mucosa results in a decreased expression of GKN1. GKN1 expression is lost in gastric cancer [72,73]. TFF2 expression is found to be markedly decreased in gastric cancer, the downregulation of which was found to be regulated by promoter hypermethylation. TFF2 has been suggested as a tumor suppressor in gastric carcinogenesis and metastasis [74,75].

The candidate proteins BAG2, GREM1, OLFM4, TRIP6, and MAGE-A9, were shown to be differentially expressed and validated using IHC in the diffuse and intestinal subtypes of gastric cancer. The *BAG* (Bcl-2-associated athanogene) family was first identified as a group of proteins that prevent cell death through their interaction with Bcl-2 [93,94]. BAG2 has been found to be overexpressed in oral squamous cell carcinoma and is associated with a poor prognosis [95]. Yue et al. found that BAG2 promotes mutant p53 accumulation and gain-of-function (GOF) mutations in tumors [96]. BAG2 binds to mutp53 and is translocated to the nucleus to inhibit the MDM2-mutp53 interaction. It also inhibits MDM2-mediated ubiquitination and degradation of mutp53. Hence, BAG2 promotes accumulation of mutp53 and GOF in the processes of tumor growth, metastasis and resistance to treatment.

It has also been shown to have a pro-oncogenic role in triple negative breast cancer cell lines [97]. Its role in gastric cancer has not been studied. Overexpression of BAG2 suggests that BAG2 might have a role in the development of early metastasis and the more aggressive disease progression seen in the diffuse subtype of gastric cancer.

Bone morphogenetic proteins (BMPs) regulate the homeostasis of the gastric epithelium by controlling the biology of the parietal cells [98]. Bone morphogenetic protein-7 (BMP7) is an independent prognostic marker in gastric cancer [99]. Gremlin1 (GREM1) is a direct antagonist of BMP 2, 4, and 7 [100]. GREM1 is thought to inhibit transforming growth factor-beta signaling by preventing ligands from binding to their receptors. Studies have shown that GREM1 is expressed in multiple malignancies, including those of the lung, skin, stomach, kidney, and testis [101]. Yamasaki et al. demonstrated that the expression of GREM1 was correlated with a shallower tumor depth, smaller tumor size, less nodal involvement, vascular invasion and a better 5-year survival rate in gastric cancer [102]. Sun et al. showed that GREM1 expression is increased in gastric cancer. They also showed that increased GREM1 expression was associated with a poorer prognosis in gastric cancer patients and GREM1 promotes proliferation and tumorigenesis of GC cells in vitro [103]. Hence, the exact role of GREM1 in gastric cancer still needs to be defined.

Olfactomedin 4 (OLFM4) has been described as a biomarker of intestinal metaplasia in the stomach [104] and a marker for stem cells in the intestine [105]. OLFM4 is involved in cell adhesion and migration [106] and also has an anti-apoptotic effect in tumor cells including gastric cancer cells [107]. OLFM4 is overexpressed in various malignancies, including pancreatic cancer, head and neck squamous cell carcinoma, lung cancer, colorectal cancer and breast cancer [108,109,110].

In gastric cancer, upregulation of OLFM4 has been shown to promote tumor progression and metastasis [111].

TRIP6 plays an important role in actin assembly, cell motility, anti-apoptotic signaling, and transcriptional regulation [112]. Many studies have shown that TRIP6 is involved in various cancers and might play an important role in tumorigenesis and metastasis [113,114,115]. The expression of TRIP6 has been shown in gastric cancer but its expression in the two subtypes—diffuse and intestinal—has not been studied. TRIP6 has been shown to have a higher expression in poorly differentiated gastric cancer characterized by intense staining on IHC, which decreases with more differentiated forms of gastric cancer [116].

Overexpression of BAG2, GREM1, OLFM4, and TRIP6 in the diffuse subtype of gastric cancer on IHC as compared to the intestinal subtype suggests a role of these proteins in the pathogenesis and progression of the diffuse subtype.

Melanoma-associated antigen A9 (MAGE-A9), is a member of the *MAGEA* gene family. Increased MAGE-A9 expression has been found to be associated with stemness and tumorigenicity in hepatocellular carcinoma [117]. Increased MAGE-A9 expression has also been associated with poor patient outcome and reduced survival in non-small cell lung cancer including squamous and adenocarcinoma, breast carcinoma and hepatocellular carcinoma [118,119,120]. Overexpression of MAGE-A9 suggests that MAGE-A9 may have a role in the development and progression of intestinal subtype of gastric cancer.

The correlation between the transcriptome and the proteome in both the subtypes did not reveal a high degree of correlation, which is in agreement with previous studies. Survival analysis did not show statistically significant differences between the diffuse and intestinal subtypes for the mRNA levels of *GREM1, BAG2, TRIP6, OLFM4* and *MAGE-A9.* It will be interesting to study this at the protein level in future studies.

Pathogenesis of gastric cancer involves a variety of molecular changes including genetic, epigenetic and dysregulation of signaling pathways. These molecular changes can act on different stages of the disease. Proteins are the functional units which control biological pathways in cells. They undergo many post translational modifications that may affect different pathways leading either to activation or inhibition of different molecules. These downstream events that occur as a result of post translational modifications may or may not play a crucial role in those pathways that eventually affect disease outcomes. Another approach to determine the role of these proteins (GREM1, BAG2, OLFM4, TRIP6 and MAGE-A9) is to inhibit their function in order to see their effect in disease progression. Further functional studies will also be needed to elucidate the exact role of these proteins in diffuse and intestinal subtypes of gastric cancer.

One important factor affecting the correlation between mRNA expression and overall survival (OS) in the TCGA dataset is that a subset of patients in both the intestinal (*n* = 34) and diffuse (*n* = 39) subtypes received adjuvant chemotherapy and/or radiotherapy [3]. The chemotherapeutic agents used, dose and duration of either chemotherapy and/or radiotherapy administered to the patients in the TCGA dataset is not available. In a study by Wu et al., where they studied the correlation between mRNA expression of B-cell lymphoma 2 interacting mediator of cell death (*BIM*), astrocyte elevated gene-1 (*AEG-1*) and AXL receptor tyrosine kinase (*AXL*) and OS in gastric cancer, no association between mRNA expression and OS for any of these genes following first-line chemotherapy with folinic acid, 5-fluorouracil (5-FU), and oxaliplatin (FOLFOX) was found [121]. However, they did notice a strong association between mRNA expression of *BIM* and OS in a subset of patients who received second-line docetaxel-based chemotherapy. Similar findings have been observed in other studies [122,123]. This shows that the association between mRNA expression and OS in gastric cancer can be affected by chemotherapy and is also dependant on the chemotherapeutic agents used. Clinical studies to examine the association of mRNA expression of *GREM1, BAG2, OLFM4, TRIP6* and *MAGE-A9* and OS in patients with intestinal or diffuse subtype of gastric cancer using different chemotherapeutic agents need to be done. The findings from these studies would provide clinically useful information that could be used to tailor treatment in gastric cancer.

## 5. Conclusions

Mass spectrometry-based proteomic profiling of diffuse and intestinal subtypes of gastric cancer was performed with the aim of identifying differences between the two subtypes at a proteomic level. A total of 4846 proteins and 7448 proteins were identified in the diffuse and intestinal subtypes, respectively. Analysis of the dysregulated proteins in both subtypes identified GREM1, BAG2, OLFM4, TRIP6 and MAGE-A9 to be differentially expressed in the diffuse subtype in comparison to the intestinal subtype. These five proteins—GREM1, BAG2, OLFM4, TRIP6 and MAGE-A9—were further validated by IHC in a larger independent cohort. Taken together, we propose that our proteomic profiling of diffuse and intestinal subtypes of gastric cancer provides a preliminary groundwork for an improved systems-level understanding of the biology and pathogenesis of the diffuse and intestinal subtypes of gastric cancer. Functional studies on these proteins might provide an insight into their mechanistic interactions in gastric cancer. The limitation of our study is that the validation cohort was relatively small, especially in the diffuse subtype.

## Figures and Tables

**Table 1:** Number of samples from the TCGA gastric cancer data belonging to the diffuse and intestinal subtypes of gastric cancer.

Gastric Cancer Subtype	Normal	Tumor
Diffuse	174	69
Intestinal	174	82

**Table 2:** Number of genes with RNA-Seq expression data from the TCGA data corresponding to the differentially expressed genes in the protein data in the diffuse and intestinal subtypes.

Data	Number of Genes	
	Diffuse	Intestinal
RNA-Seq	578	69
Protein	610	70

**Table 3:** Baseline demographic and disease characteristics for patients in the discovery cohort. AD—Adenocarcinoma, DG—Distal gastrectomy, F—Female, M—Male, RG—Radical gastrectomy, SA—South Asian, SRCC—Signet ring cell carcinoma.

Characteristic	Diffuse					Intestinal				
Patients	1	2	3	4	5	1	2	3	4	5
Age (years)	66	50	65	65	60	48	60	55	64	53
Sex	M	M	M	M	F	M	M	M	M	F
Ethnicity	SA	SA	SA	SA	SA	SA	SA	SA	SA	SA
Surgical procedure	DG	DG	DG	DG	DG	DG	DG	DG	RG	RG
Histopathologic diagnosis	SRCC	SRCC	SRCC	SRCC	SRCC	AD, Grade 2	AD, Grade 3	AD, Grade 2	AD, Grade 2	AD, Grade 3

**Table 4:** A partial list of differentially expressed proteins in diffuse subtype of gastric cancer.

Gene	Protein	Fold-Change (Relative Abundance as Compared to the Normal)
*VCAN*	Versican core protein	12.7
*COL6A3*	Collagen alpha-3(VI) chain	8.6
*NCBP2-AS2*	Uncharacterized protein NCBP2-AS2	8.4
*HOPX*	Homeodomain-only protein	8.2
*COL16A1*	Collagen alpha-1(XVI) chain	7.6
*SFRP4*	Secreted frizzled-related protein 4	7.2
*FCN3*	Ficolin-3	6.7
*CNN1*	Calponin-1	6.2
*MXRA7*	Matrix-remodeling-associated protein 7	6.1
*GUCY1B1*	Guanylate cyclase soluble subunit beta-1	6.1
*GHITM*	Growth hormone-inducible transmembrane protein	0.5
*CWF19L1*	CWF19-like protein 1	0.5
*MAOA*	Amine oxidase [flavin-containing] A	0.5
*BCS1L*	Mitochondrial chaperone BCS1	0.5
*CISD3*	CDGSH iron-sulfur domain-containing protein 3	0.5
*SCO1*	Protein SCO1 homolog, mitochondrial	0.5
*NDUFB10*	NADH dehydrogenase [ubiquinone] 1 beta subcomplex subunit 10	0.5
*GCSH*	Glycine cleavage system H protein	0.5
*NCLN*	Nicalin	0.5
*PRDX3*	Thioredoxin-dependent peroxide reductase	0.5

**Table 5:** A partial list of differentially expressed proteins in intestinal subtype of gastric cancer.

Gene	Protein	Fold-Change (Relative Abundance as Compared to the Normal)
*SLAIN2*	SLAIN motif-containing protein 2	2.7
*HMCN1*	Hemicentin-1	2.3
*SCML2*	Sex comb on midleg-like protein 2	2.3
*SERPINE1*	Plasminogen activator inhibitor 1	2.3
*SPARC*	SPARC	2.3
*P3H3*	Prolyl 3-hydroxylase 3	2.2
*IGFBP7*	Insulin-like growth factor-binding protein 7	2.2
*BDP1*	Transcription factor TFIIIB component B” homolog	2.2
*SULF1*	Extracellular sulfatase Sulf-1	2.2
*ABCA1*	ATP-binding cassette sub-family A member 1	2.2
*RTN4*	Reticulon-4 isoform C	0.5
*FAAH*	Fatty-acid amide hydrolase 1	0.5
*ZG16B*	Zymogen granule protein 16 homolog B	0.5
*ATP4A*	Potassium-transporting ATPase alpha chain 1	0.5
*FRMD4B*	FERM domain-containing protein 4B	0.5
*ALDH3A1*	Aldehyde dehydrogenase, dimeric NADP-preferring	0.5
*COL9A1*	Collagen alpha-1(IX) chain	0.5
*REG3A*	Regenerating islet-derived protein 3-alpha	0.5
*SULT1E1*	Estrogen sulfotransferase	0.5
*PBLD*	Phenazine biosynthesis-like domain-containing protein	0.5

## Data Availability

The mass spectrometry proteomics data have been deposited to the Proteome Xchange Consortium via the PRIDE partner repository with the dataset identifier PXD026279.

## Supplementary Materials

The following are available online at https://www.mdpi.com/article/10.3390/cancers13235930/s1, Table S1: List of fold-change values for RNA-Seq expression data and corresponding proteins for diffuse and intestinal subtypes, Table S2: Details of parameters used for survival analysis using Kaplan-Meier curves for the diffuse (D) and intestinal (I) subtypes, Table S3: List of the proteins identified in both the intestinal and diffuse subtypes of gastric cancer along with their fold-change values, Table S4: H-score values for semi-quantitative assessment of IHC staining of GREM1, BAG2, TRIP6, OLFM4 and MAGE-A9 in the validation set of cases of diffuse and intestinal subtypes of gastric cancer, Table S5: List of the top five canonical pathways identified in diffuse and intestinal subtypes with the proteins involved in each pathway and their corresponding fold-change values, Figure S1: Top network in diffuse subtype of gastric cancer predicted by IPA. The network is graphically displayed with genes/gene products as nodes (different shapes represent the functional classes of the gene products as indicated in the legend) and the biological relationships between the nodes as edges (lines). The length of an edge reflects the evidence in the literature supporting that node-to-node relationship. The color intensity of the nodes indicates the degree of up- (red) or downregulation (green) of the respective protein, Figure S2: Top network in the intestinal subtype of gastric cancer predicted by IPA. The network is graphically displayed with genes/gene products as nodes (different shapes represent the functional classes of the gene products as indicated in the legend) and the biological relationships between the nodes as edges (lines). The length of an edge reflects the evidence in the literature supporting that node-to-node relationship. The color intensity of the nodes indicates the degree of up- (red) or downregulation (green) of the respective protein, Figure S3. The association between mRNA expression levels of *GREM1* (A), *BAG2* (B), *OLFM4* (C), *TRIP6* (D) and *MAGE-A9* (E) from the TCGA dataset and overall survival in diffuse or intestinal subtypes is shown (Kaplan–Meier analysis, *p*-value from log-rank test, Diffuse subtype-blue, Intestinal subtype-green).
