# Supplementary material for: Proteomic Signatures of Diffuse and Intestinal Subtypes of Gastric Cancer
Source: Cancers (Basel). 2021 Nov 25;13(23):5930. doi: 10.3390/cancers13235930 (PMC8656738; doi:10.3390/cancers13235930)
Supplement: Supplementary file 1 [file cancers-13-05930-s001.zip › Updated Supplementary material_111921/Supplementary Table S4_H-score values_072621.pdf]

Table S4. H-score values for semi-quantitative assessment of IHC staining of GREM1, BAG2, TRIP6, OLFM4 and MAGE-A9 in the validation set of cases of diffuse and intestinal subtypes of gastric cancer

**Gastric cancer subtype**	**H-score (Average)**				
	GREM1	BAG2	TRIP6	OLFM4	MAGE-A9
Diffuse (n=16)	201.6	169.4	175.2	187.4	31.9
Intestinal (n=108)	32.9	28.9	27.2	26	144.8
