# Supplementary figures and images for: Proteomic Signatures of Diffuse and Intestinal Subtypes of Gastric Cancer
Source: Cancers (Basel). 2021 Nov 25;13(23):5930. doi: 10.3390/cancers13235930 (PMC8656738; doi:10.3390/cancers13235930)

**Figure S1**

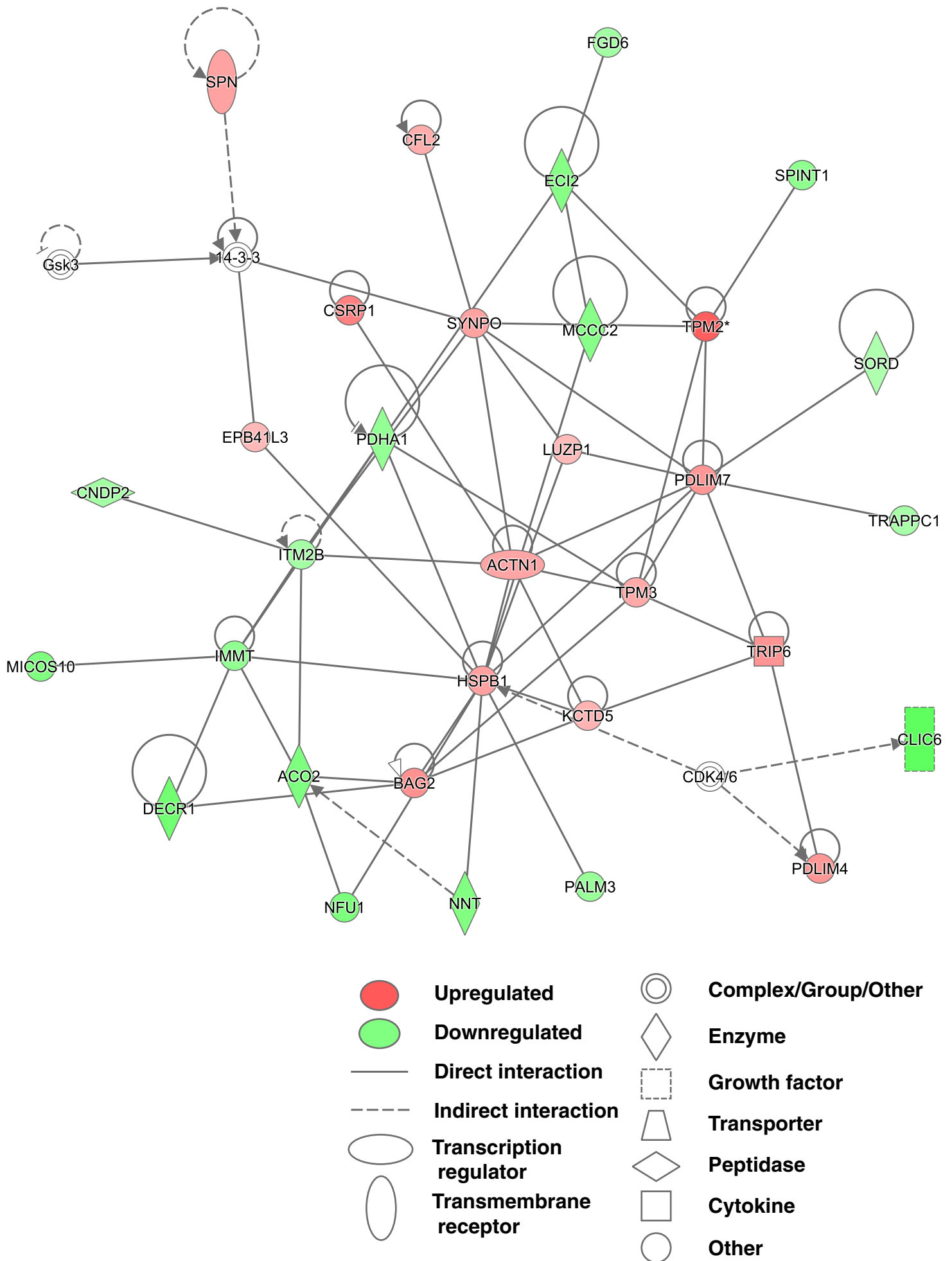

Supplement: Supplementary file 1 [file cancers-13-05930-s001.zip › Updated Supplementary material_111921/Figure S1 DIFFUSE_IPA_Network_091621.pdf]

**Figure S2**

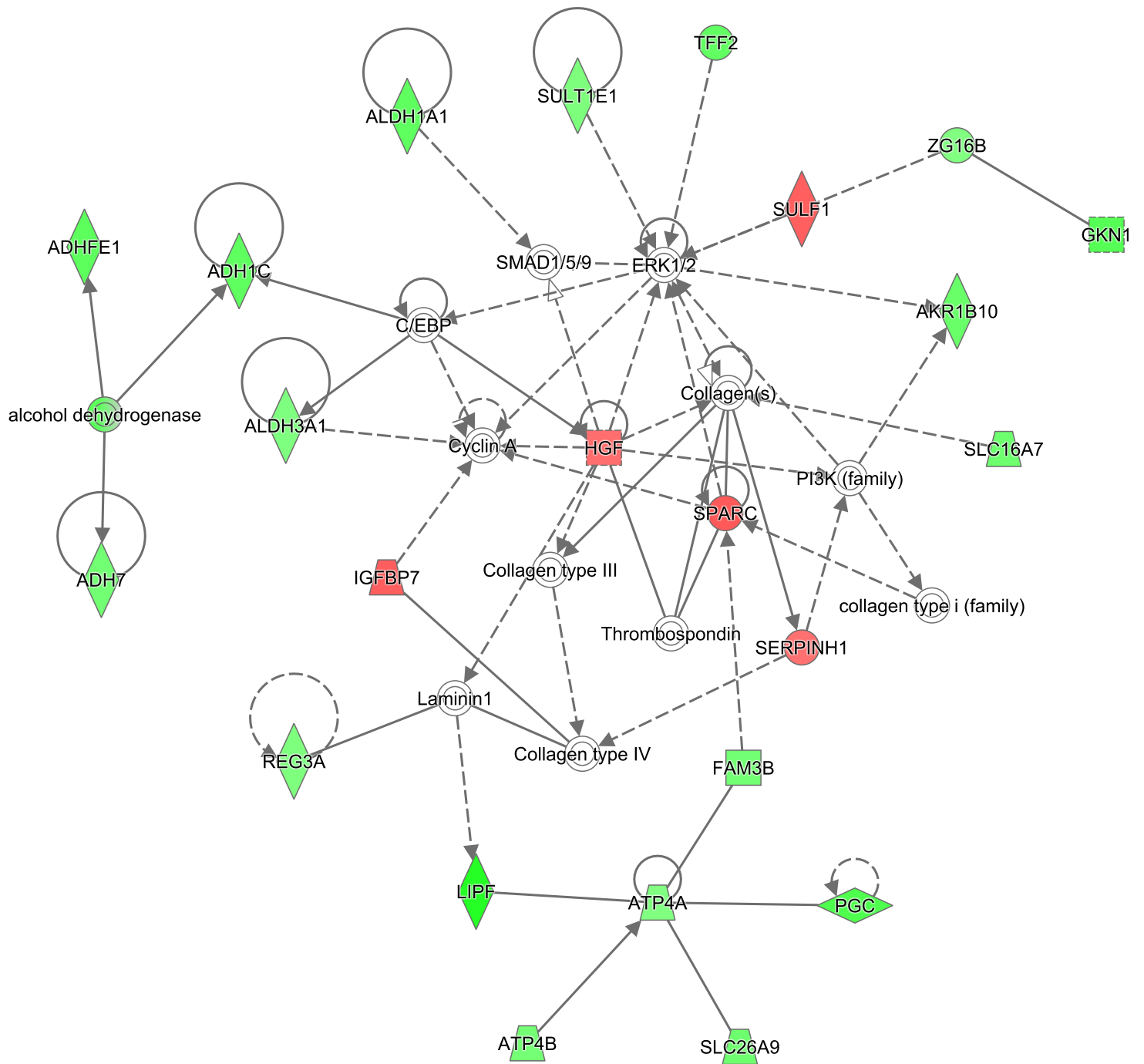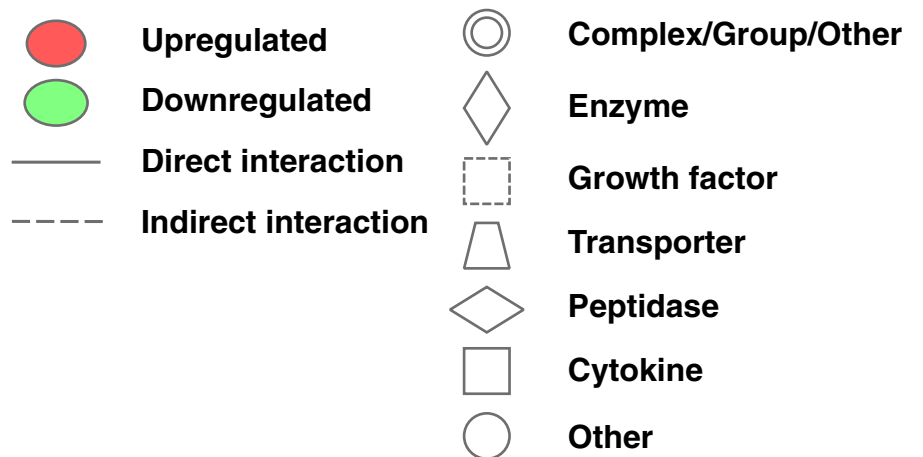

Supplement: Supplementary file 1 [file cancers-13-05930-s001.zip › Updated Supplementary material_111921/Figure S2 Intestinal_network_IPA_091621.pdf]

**Figure S3**

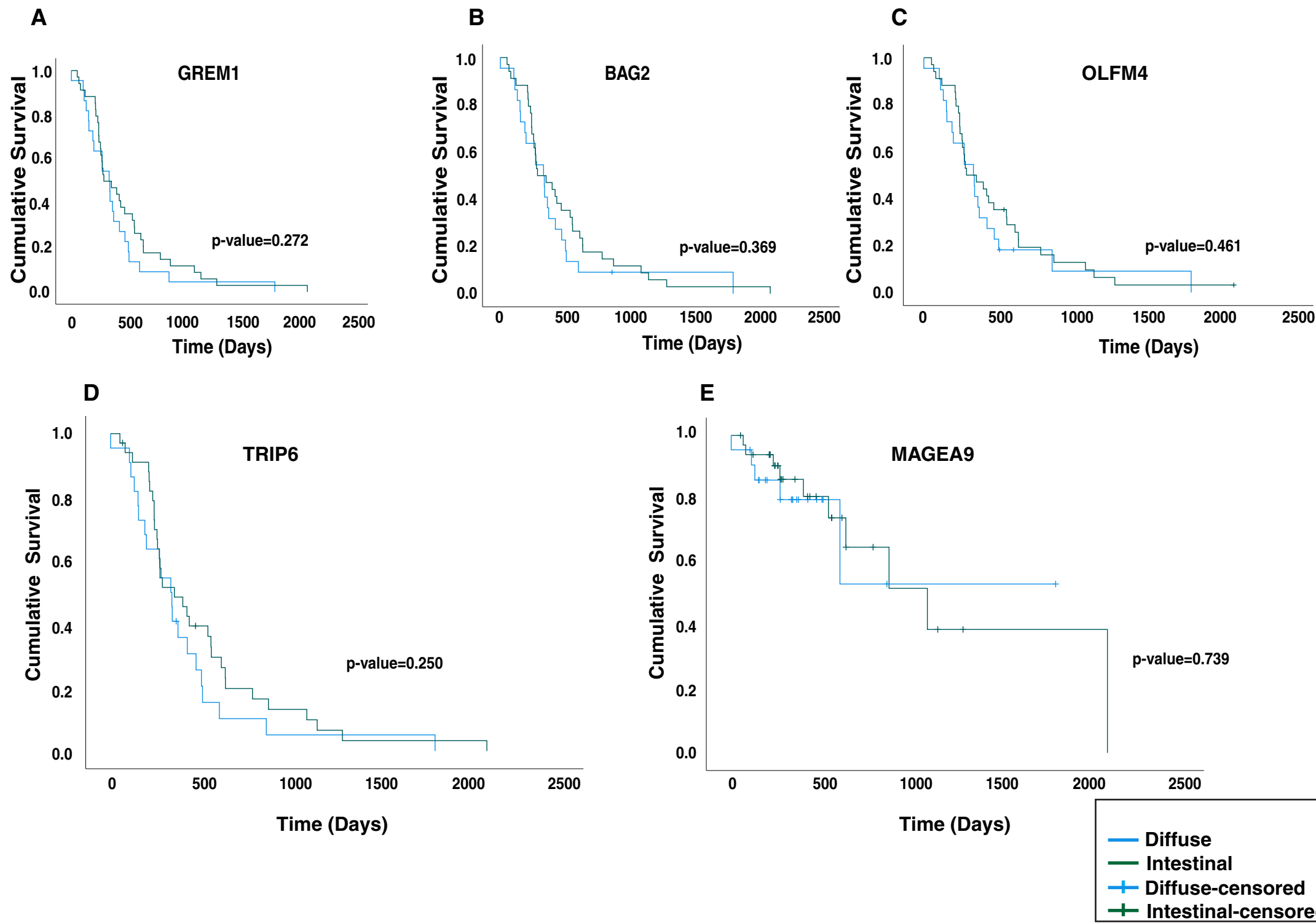

Supplement: Supplementary file 1 [file cancers-13-05930-s001.zip › Updated Supplementary material_111921/Figure S3_111721.pdf]
